# The role of intention and self-efficacy on the association between breastfeeding of first and second child, a Danish cohort study

**DOI:** 10.1186/s12884-018-2086-5

**Published:** 2018-11-22

**Authors:** Hanne Kronborg, Else Foverskov, Michael Væth, Rikke D. Maimburg

**Affiliations:** 1 Department of Public Health, Section for Nursing, Aarhus University, 8000 Aarhus C, Denmark; 2 Department of Public Health, Section of Social Medicine, University of Copenhagen, Copenhagen, Denmark; 3 Department of Public Health, Section for Biostatistics, Aarhus University, Aarhus C, Denmark; 4 Department of Clinical Medicine, Aarhus University, Aarhus, Denmark; 5 Department of Obstetrics and Gynaecology, Aarhus University Hospital, Aarhus, Denmark; 6 Centre of Research in Rehabilitation (CORIR), Aarhus University Hospital, Aarhus, Denmark

**Keywords:** Parity, breastfeeding, Self-efficacy and intention, Follow-up study, Statistics & numerical data

## Abstract

**Background:**

The impact of parity on breastfeeding duration may be explained by physiological as well as psychosocial factors. The aim in the present study was to investigate the mediating influence of intention and self-efficacy on the association between the breastfeeding duration of the first and the following child.

**Methods:**

A 5-year Danish cohort study with data from online questionnaires was used. Data came from 1162 women, who participated in the “Ready for child” trial in 2006–7 and gave birth to their second child within 5 years in 2011–3. Analysis included multiple regression models with exclusive/any breastfeeding duration of first child as the exposure variables, intention and self-efficacy measured as mediators, and exclusive/any breastfeeding duration of the second child as the outcome variables.

**Results:**

Duration of exclusive breastfeeding of the first child was significantly associated with exclusive breastfeeding duration of the second child (*p* <  0.001) and with the self-reported intention and self-efficacy in the ability to breastfeed the second child (*p* <  0.001). The exclusive breastfeeding period was slightly longer for the second child. Self-efficacy and intention mediated the association between breastfeeding duration in the first and second child. Together the two factors explained 48% of the association in exclusive breastfeeding and 27% of the association in any breastfeeding between the first and second child.

**Conclusion:**

Due to a reinforcing effect of intention and self-efficacy, breastfeeding support should focus on helping the first time mothers to succeed as well as to identify the second time mother with low self-efficacy and additional need for support.

**Electronic supplementary material:**

The online version of this article (10.1186/s12884-018-2086-5) contains supplementary material, which is available to authorized users.

## Background

Breastfeeding rates are only increasing slowly in Western societies [1] even though exclusive breastfeeding has been the recommended nutrition for the first 6 months of life since 2001 [2]. The ideal nutritional composition of breast milk leads to optimal infant growth as well as positive short and long term health benefits [3]. Support from professionals or lay persons to mothers who have initiated breastfeeding have shown positive effect on reducing the risk of early cessation of exclusive breastfeeding [4]. Breastfeeding duration is influenced by multiple factors related to both the mother and the infant. Infant characteristics such as lower gestational age and birth weight have been shown to be related to shorter breastfeeding duration [5, 6]. Furthermore, maternal demographic and socioeconomic characteristics such as lower maternal age, educational level, and social status, smoking and high BMI are well-known factors related to earlier breastfeeding cessation [7]. In the last decades the focus has in addition been on the role of psychosocial factors such as intention to breastfeed and self-efficacy or maternal confidence of breastfeeding as contributory explanations for early cessation of breastfeeding in Western societies [12, 13].

Several studies have reported how breastfeeding duration of the first child has been found to be predictive for breastfeeding duration in following child [16–18]. The impact of parity on breastfeeding duration may be due to both physiological as well as psychosocial factors. Physiologically, the multipara woman has an earlier onset of lactation [19], which may influence the success of early lactation [20]. Psychosocially, the multipara woman has positive or negative breastfeeding experiences from the first child, which may influence her intention and self-efficacy towards breastfeeding her next child. The extent to which psychosocial factors can explain why the breastfeeding duration for the first child is predictive for the duration of breastfeeding the second child is scarcely investigated. However, to be able to support the multipara mother to succeed in breastfeeding, we need to know more about the mechanisms that link previous breastfeeding experiences with the breastfeeding duration of the following child [16, 21, 22]. Thus, the aim of the present study was to examine the association between the breastfeeding duration of the first and the second child in a cohort of Danish women. Moreover, to investigate if an association could be explained by the effect the breastfeeding duration of the first child may have had on mothers’ intentions and self-efficacy to breastfeed their second child.

## Methods

### Design and participants

A cohort study design was used following Danish women who participated in the “Ready for child” trial over 5 years [23, 24]. Women were recruited from the Aarhus Midwifery Clinic located at a university hospital in an urban area of Denmark. From May 2006 to May 2007 all pregnant women, who registered for antenatal care, were invited to participate. The invitation included oral and written information about the study. Women were enrolled from week 10 + 0 to week 21 + 6 of gestation. Inclusion criteria were nullipara, older than 18 years of age at enrollment, with a singleton pregnancy, and the ability to speak and understand Danish. Before enrolment, informed consent was obtained from all participating women including a written permission to send out questionnaires for long term follow-up. When pregnant with their first child in 2006–07, the women were randomized to receive either 9 h of formalised antenatal care focusing on the birth process, breastfeeding, and parenting skills or to receive standard care. The main results from the previous trial are reported elsewhere [23, 25]. In the month where their first child turned 5 years in 2011–13 a letter including information about the follow-up online questionnaire was sent to the women. No letter was sent to previous participants who were deceased, had emigrated or had an unknown address. The number of previous participants who had given birth to their second child and completed the follow-up questionnaire determined the sample size for the study.

### Data-collection and variables

The data were obtained from three questionnaires forwarded to the participating mothers at 6 weeks, 1 and 5 years after giving birth to their first child [23, 25]. Furthermore, obstetric information on the first and the second child was retrieved from a local birth cohort database (the Aarhus Birth Cohort). This database includes information validated by trained midwives. One year postpartum questionnaire data were collected on breastfeeding duration of the first child as well as breastfeeding intention and self-efficacy concerning a potential second child. In the 5-year follow-up questionnaire, retrospective data were collected on experiences and duration of breastfeeding the second child. Questionnaires can be accessed online in Additional file 1.

***The outcome variables*** were duration of exclusive breastfeeding and duration of any breastfeeding of the second child.

***The exposure variables*** were duration of exclusive breastfeeding and duration of any breastfeeding of the first child. All variables were collected according to indicators for assessing breastfeeding practices [26] by asking the mother: “How many weeks did you breastfeed your first / second child without giving any supplement at all except from on single occasions?” (exclusive breastfeeding), and “how many weeks were your first / second child when you stopped breastfeeding entirely?” (any breastfeeding).

***Mediators*** addressing the psychosocial aspect of breastfeeding were collected by asking the mothers 1 year postpartum on intention and self-efficacy to breastfeed a potential second child. The variables were measured reflecting the conceptual framework of Fishbein and Ajzen’s Theory of Reasoned Action [27] and Bandura’s Social Cognitive Theory [28]. Intention was measured in weeks by asking the mothers: “How many weeks do you intend to breastfeed your next infant?”. The overall self-efficacy was measured situation- and domain-specific on a five-point Likert scale from very certain to very uncertain by asking the mother: “how certain are you to carry on with breastfeeding your next infant until he/she reaches the age of 4 months?”

***Covariates*** included maternal socio-demographic characteristics at the time of the birth of the first child and the second child’s birth weight and gestational age as well as perinatal conditions and perceptions of postnatal care from the hospital and the health visitor. All variables with sub-categories appear from Table 1.

**Table 1:** Bivariate associations between study variables and breastfeeding duration of the second child, one-way ANOVA and Pearson’s correlation

Variable	Exclusive breastfeeding duration second child (*N* = 717)				Any breastfeeding duration second child (*N* = 691)			
	% missing	Mean	SD	*P*-value	% missing	Mean	SD	*P*-value
Breastfeeding experience (1st child)								
Duration of exclusive breastfeeding								
Less than 2 months	5.58	11.29	11.09	< 0.001				
2–4 months		15.24	7.25					
4–6 months		20.55	6.89					
More than 6 months		21.64	10.19					
Duration of any breastfeeding								
Less than 4 months					6.22	17.37	14.22	< 0.001
4–8 months						29.58	11.79	
8–12 months						37.70	11.55	
More than 12 months						48.79	13.83	
Duration of exclusive breastfeeding (weeks)								
Correlation	5.58	*0.418*		< 0.001				
Duration of any breastfeeding (weeks)								
Correlation					6.22	*0.595*		< 0.001
Breastfeeding intentions and self-efficacy (2nd child)								
Intended breastfeeding duration (weeks)								
Correlation	4.88	*0.352*		< 0.001	4.63	*0.511*		< 0.001
Confidence in ability to breastfeed for 4 months								
Certain	4.74	19.68	7.84	< 0.001	4.49	37.05	14.10	< 0.001
Neither nor / Uncertain		10.05	9.78			20.56	16.57	
Maternal factors								
Age (years)								
Correlation	0.00	*0.054*		0.150	0.00	*0.059*		0.121
Educational level								
Low level	0.00	17.08	10.00	0.163	0.00	30.77	16.56	0.012
Mid-level		17.74	9.20			33.92	15.12	
High level		18.80	8.47			36.00	15.89	
Perinatal factors (2nd child)								
Gender								
Boy	0.00	17.63	9.68	0.175	0.00	33.59	16.10	0.232
Girl		18.55	8.31			35.03	15.33	
Gestational age at birth (weeks)								
Correlation	0.00	*0.100*		0.007	0.00	*0.067*		0.080
Birth weight (kg)								
Correlation	0.14	*0.010*		0.790	0.14	*0.028*		0.462
Caesarean section								
Yes	0.00	16.21	9.72	0.009	0.00	31.48	17.71	0.030
No		18.49	8.84			34.88	15.23	
Formula supplement, hospital								
Yes	0.00	14.05	10.56	< 0.001	0.00	30.60	18.27	0.003
No		19.02	8.38			35.16	14.97	
Breastfeeding support (2nd child)								
Received the necessary support for breastfeeding in the hospital								
Yes	1.95	18.67	8.87	0.013	2.03	35.22	15.14	0.031
Neither nor / No		16.74	9.17			32.23	17.23	
Received the necessary support for breastfeeding from the health visitor								
Yes	2.51	18.43	8.90	0.195	2.60	34.71	14.99	0.337
Neither nor / No		17.46	9.25			33.44	17.13	
Received the necessary support for breastfeeding from the father								
Yes	0.42	18.26	8.93	0.197	0.43	34.50	15.48	0.463
Neither nor / No		16.39	9.90			32.67	18.57	
Intervention programme								
Control group	0.00	17.55	9.82	0.146	0.00	35.48	14.99	0.033
Intervention group		18.54	8.28			32.92	16.47	

### Statistical analysis

Bivariate associations between all variables and breastfeeding duration (exclusive and any) of the second child were assessed with one-way ANOVA for categorical variables and Pearson’s correlation coefficient for continuous variables (Table 1). The association between breastfeeding duration of the first child and the mediators were examined using linear regression in the case of intention and logistic regression in the case of self-efficacy (Table 2). In both cases the associations were adjusted for maternal age and educational level. Measures of maternal educational level only changed slightly between baseline and follow-up and the baseline values were therefore used in all analyses. Linear regression models were applied to compute the association between breastfeeding duration of the first and second child when controlling for covariates (Table 3, Model 1). Models were estimated for both exclusive and any breastfeeding duration. These models provided an estimate of the total impact of breastfeeding duration of the first child on breastfeeding duration of the second child. Subsequently, the same models were computed including the mediators to get an estimate of the direct influence of breastfeeding duration of the first child on breastfeeding duration of the second child, not mediated by the measures of intention and self-efficacy (Table 3, Model 2 and 3). Finally, the difference between the direct and the total influence (the indirect influence) was calculated to get an estimate of how much of the association between breastfeeding duration of the first and second child acted through the mediators. The mediated proportion was derived by dividing the indirect influence by the total influence and a 95% confidence interval around this proportion was estimated using bootstrapping with 500 replications. This procedure of studying mediation followed the approach by Baron and Kenny [29]. Missing data were handled using listwise deletion. STATA 14 was used for all statistical analyses [30].

**Table 2:** Associations between breastfeeding duration of the first child and intention to breastfeed the second child from linear regression models (coefficients and 95% confidence intervals) and between breastfeeding duration of the first child and self-efficacy to breastfeed the second child from logistic regression models (odds ratios and 95% confidence intervals)

	Intended breastfeeding duration 2nd child, weeks		Confidence in ability to breastfeed 2nd child for 4 months	
	Coef.	95% CI	OR	95% CI
Exclusive breastfeeding 1st child, weeks (*N* = 699)	0.64^***^	[0.54, 0.75]	1.22^***^	[1.18, 1.26]
Any breastfeeding 1st child, weeks (*N* = 694)	0.69^***^	[0.64, 0.74]	1.13^***^	[1.11, 1.16]

^***^p < 0.001

Adjusted for maternal age and educational level

**Table 3:** Associations from linear regression models (coefficients and 95% confidence intervals) between breastfeeding duration in the first and the second child unadjusted and adjusted for intention and self-efficacy

	Model 1		Model 2		Model 3	
	Coef.	95% CI	Coef.	95% CI	Coef.	95% CI
Outcome: Exclusive breastfeeding (weeks), *N* = 650						
Duration of exclusive breastfeeding, first child (weeks)	0.36^***^	[0.29, 0.44]	0.28^***^	[0.20, 0.36]	0.19^***^	[0.10, 0.27]
Intended breastfeeding duration, second child (weeks)			0.14^***^	[0.09, 0.19]	0.12^***^	[0.07, 0.17]
Confidence in ability to breastfeed for 4 months, second child					4.69^***^	[2.69, 6.69]
Outcome: Any breastfeeding (weeks), *N* = 623						
Duration of any breastfeeding, first child (weeks)	0.65^***^	[0.58, 0.73]	0.53^***^	[0.42, 0.63]	0.48^***^	[0.37, 0.59]
Intended breastfeeding duration, second child (weeks)			0.19^**^	[0.08, 0.29]	0.19^**^	[0.08, 0.30]
Confidence in ability to breastfeed for 4 months, second child					3.60^*^	[0.38, 6.82]

^*^p < 0.05; ^**^p < 0.01; ^***^p < 0.001

Adjusted for maternal age, maternal educational level, gender, gestational age, birth weight, caesarean section, formula supplement in hospital, received support in the hospital, received support from the health visitor, received support from the father and intervention

## Results

**Fig. 1:**
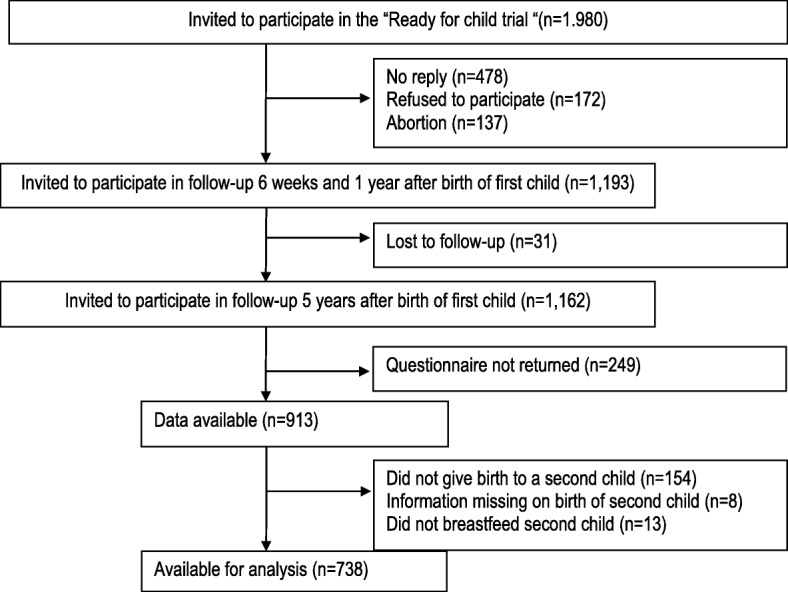
Flow profile and exclusion criteria for selection of population available for analysis

The “Ready for child” trial was initiated in 2006–07 and 1980 women pregnant with their first child were invited to participate; 60% agreed to be included in the study. Between data collection at 6 weeks and 1 year after the birth of their first child, 31 participants were lost to follow-up, leaving 1162 women for the follow-up 5 years postpartum. The response rate at the 5-year follow-up was 79%. Among the respondents, 154 women who had not given birth to a second child during this 5-year period, eight women who had missing information on whether they had given birth to a second child, and 13 women who did not initiate breastfeeding of their second child were excluded. This resulted in a population of 738 women available for analysis (Fig. 1).

Table 1 shows bivariate associations between the study variables and exclusive and any breastfeeding duration of the second child as well as the proportion of missing data for the study variables. The proportion of missing data was highest for the exposure variables on breastfeeding experience (5–6%). The exclusive breastfeeding period was longer for the second child compared to the first child, especially in mothers who had exclusively breastfed their first child for less than 4 months. The exclusive breastfeeding period was shorter for the second child among mothers who had exclusively breastfed their first child for more than 4 months. Duration of any breastfeeding followed the same pattern as exclusive breastfeeding. The number of weeks of breastfeeding the first and second child was significantly correlated (*p* <  0.001) with a coefficient of 0.42 for exclusive breastfeeding and 0.60 for any breastfeeding. The intention and self-efficacy in relation to breastfeeding the second child reported when the first child was 1 year of age were significantly associated with the duration of exclusive and any breastfeeding of the second child (*p* <  0.001). Socio-demographic factors were not associated with breastfeeding duration, except for maternal educational level which was significantly correlated with the duration of any breastfeeding (*p* = 0.01). Among the perinatal factors, lower gestational age in the second child, undergoing a cesarean section or infants receiving formula supplement in hospital were significantly correlated with shorter duration of exclusive breastfeeding (*p* <  0.01) and shorter duration of any breastfeeding if the infant was delivered by cesarean section (*p* = 0.03) or had received formula supplement (*p* <  0.01). Gender and birth weight of the second child were not associated with breastfeeding duration. Maternal self-reported support received in relation to breastfeeding in the hospital was associated with breastfeeding duration (*p* = 0.01 for exclusive breastfeeding and *p* = 0.03 for any breastfeeding). However, the self-reported support received from the health visitor or the father was not significantly associated with breastfeeding duration.

Table 2 shows how breastfeeding duration of the first child was associated with intention and self-efficacy when adjusted for maternal age and educational level. The number of weeks mothers intended to breastfeed their second child was significantly associated with both the number of weeks of exclusive breastfeeding (β = 0.64, 95% CI = 0.54, 0.75) and any breastfeeding (β = 0.69, 95% CI = 0.64, 0.74) of the first child. Longer breastfeeding durations of the first child was additionally associated with higher odds of mothers being confident in their ability to breastfeed a second child for 4 months (exclusive breastfeeding: β = 1.22, 95% CI = 1.18, 1.26 and any breastfeeding: β = 1.13, 95% CI = 1.11, 1.16).

The linear regression coefficients in Table 3 show that the duration in weeks of exclusive breastfeeding of the first child was positively associated with the duration of exclusive breastfeeding of the second child (β = 0.36, 95% CI = 0.29, 0.44) when adjusted for covariates (Model 1). When additionally controlling for the intended breastfeeding duration of the second child, the coefficient dropped to 0.28 (95% CI = 0.20, 0.36) (Model 2). When also controlling for maternal self-efficacy in the ability to breastfeed the second child for 4 months, the coefficient dropped to 0.19 (95% CI = 0.10, 0.27) (Model 3). Overall, intention and self-efficacy could explain 48% (β = 0.48, 95% CI = 0.26, 0.70) of the association between exclusive breastfeeding duration of the first and second child. For any breastfeeding the association between the duration of the first and the second child showed a total influence of 0.65 (95% CI = 0.58–0.73) (Model 1) and a direct influence of 0.48 (95% CI = 0.37–0.59) (Model 3). The mediating measures of intention and self-efficacy hereby explained 27% (β = 0.27, 95% CI = 0.11, 0.42) of the association between the duration of any breastfeeding of the first and second child.

At the 5-year follow-up after the birth of their first child, 11 mothers were still exclusively breastfeeding their second child and 36 mothers were still partially breastfeeding their second child. These mothers were excluded from the main analysis. However, supplementary analyses were carried out including these mothers, first with the current number of weeks they had exclusively or partially breastfed their second child at the 5-year follow-up and subsequently adding 4 weeks to the current number of weeks. Results from these additional analyses were not found to differ noteworthy from the results of the main analysis.

Data on breastfeeding duration of the first child were obtained from questionnaires at 6 weeks and 1 year postpartum. Thus, the duration of any breastfeeding of the first child was censored at 52 weeks. A total of 126 mothers were still partially breastfeeding their first child 1 year postpartum. Additional analyses were carried out to assess the impact of the censoring. The duration of any breastfeeding in the first child was coded in five levels (0–12 weeks, 13–25 weeks, 26–38 weeks, 39–51 weeks and 52+ weeks) and included in the analysis as a continuous variable ranging from 1 through 5, or with 6 weeks added to the start of the interval (6, 19, 32, 45 and 58). Results from these additional analyses without censoring did not differ significantly from the results of the main analysis.

## Discussion

This study found breastfeeding duration of the first and second child was highly correlated. Breastfeeding intention and self-efficacy towards future breastfeeding reported after the first breastfeeding period played a significant role in explaining the relation between breastfeeding duration of the first and second child. Intention and self-efficacy combined explained 48% of the association between exclusive breastfeeding duration of the first and second child and 27% of the association between any breastfeeding duration of the first and second child.

In the present study we used a prospective design and by collecting data at different time points we were able to disentangle the directions of the relations between the exposure, mediators and outcome variables. The reporting of the breastfeeding duration was collected retrospectively in the 5-year follow-up questionnaire, which may give risk of recall bias. On the other hand, breastfeeding duration is usually well recalled by the mothers [32] and the rates of breastfeeding found in the present study were similar to breastfeeding rates found in others studies in the same period in Denmark [32, 33]. In the multivariate analysis we adjusted for a number of co-variables. Although the maternal socio-demographic variables of age and education did not play a significant role in explaining the exclusive breastfeeding period of the second child, these have well known confounding capacity in a breastfeeding context. We therefore adjusted for these factors together with perinatal and infant-related factors. The results were robust to censoring in a number of supplementary sensitivity analysis. However, unbiased estimation of direct and indirect effects is based on an assumption of no unmeasured confounding of the outcome-mediator, mediator-exposure and outcome-exposure relationships and although we controlled for a number of time dependent factors, bias due to unmeasured confounding is a possibility.

The correlations between the duration of exclusive or any breastfeeding of the first and the second child are consistent with earlier findings from Scandinavia [11, 21, 33], Europe [14, 16, 22], USA [17], and Asia [18]. Despite national initiatives in Denmark to prolong breastfeeding duration, the pattern to repeat breastfeeding practices from the first to the second child does not seem to have changed decisively during the last two decades [34]. The present study suggests that parity as a single factor does not explain the breastfeeding duration of the second child. We found instead that the breastfeeding duration of the first child as well as the intention and self-efficacy reported after having breastfed the first child were strongly associated with the duration of exclusive breastfeeding of the second child.

The psychosocial factors intention and self-efficacy [35] have earlier been found to be the most influential predictors for breastfeeding duration [12, 37]. To the best of our knowledge, the present study demonstrated for the first time how intention and self-efficacy concerning breastfeeding a future child mediate the association between breastfeeding duration of the first and second child. The two factors explained nearly half of the association in the case of exclusive breastfeeding. The questions regarding the self-reported intention and self-efficacy reflected a conceptual framework with components of Ajzen and Fishbein’s theory of Reasoned Action [28] and Banduras Social Cognitive theory [37]. According to the grounded theories, intention is the most influential factor on future behavior and self-efficacy forms your beliefs in being able to perform in the new situation. Second time mothers who have had limited success with breastfeeding their first child may be affected by having a lower intention and self-efficacy in breastfeeding the next child. A low self-efficacy towards a task, in this case breastfeeding, may according to Bandura result in a person losing perspective and being more likely to give up, especially if early problems turn up, which is often the case in breastfeeding [38]. An important target group for supplemental breastfeeding support may therefore be second time mothers with previous short breastfeeding experience [39]. Especially in the early period following birth where unexpected breastfeeding problems relatively often occur and may pressure the second time mothers with relatively low self-efficacy to give up breastfeeding. Prevention may consist of an early screening among second time mothers to identify low self-efficacy and the need for additional breastfeeding support [40]. Breastfeeding support provided to second time mothers with low self-efficacy could include components suggested by Bandura such as skills training with guided practice and feedback, modelling, identification of high-risk situations with planning of coping responses, and mobilizing social support [41]. Our findings also clarify the importance of preparing and supporting the first time mother and thereby assist her in building positive intentions and self-efficacy towards breastfeeding future children. According to the newly updated Cochrane review, positive impact of breastfeeding support includes face-to-face support and a scheduled ongoing contact with healthcare professionals [4].

Another interesting finding was that the proportion of mothers who had previously breastfed their first child for less than 4 months showed a tendency to breastfeed exclusively for a longer period (increase) whereas mothers who had exclusively breastfed their first child for more than 4 months showed a tendency to exclusively breastfeed for a shorter period (decrease) the second time. Similar patterns have earlier been found by Nagy et al. [22] among Hungarian mothers concerning any breastfeeding and by Bay et al. [18] among Hong Kong mothers concerning exclusive breastfeeding. The reason for this tendency is unknown. It may simply be due to regression to the mean or it may derive from calculated considerations among second time mothers. Further studies are needed to explain the phenomenon if we choose not only to regard it as a regression to the mean.

This study was performed in Denmark, Scandinavia where the proportion of mothers who breastfeed is high compared to other western countries. Still, the influence of psychosocial factors on breastfeeding duration have been found to be consistent in western societies during the last decades [12, 13]. Therefore, we consider our results generalizable to other western societies with the limitation that the influence of cultural aspects are unknown.

## Conclusion

Previous breastfeeding duration, which may be an expression of earlier breastfeeding experience, is strongly associated with the intention and self-efficacy of breastfeeding a future child. The two psychosocial factors intention and self-efficacy explain nearly half of the association between the breastfeeding durations of the first and the second child and are therefore important to consider when planning and conducting future breastfeeding support.

## Additional file

Additional file 1: Questions used in the study: the role of intention and self-efficacy on the association between breastfeeding of first and second child, a Danish cohort study. (DOCX 25 kb)
