# Supplementary material for: The role of intention and self-efficacy on the association between breastfeeding of first and second child, a Danish cohort study
Source: BMC Pregnancy Childbirth. 2018 Nov 22;18:454. doi: 10.1186/s12884-018-2086-5 (PMC6251224; doi:10.1186/s12884-018-2086-5)
Supplement: Supplementary file 1 — Questions used in the study: the role of intention and self-efficacy on the association between breastfeeding of first and second child, a Danish cohort study. (DOCX 25 kb) [file 12884_2018_2086_MOESM1_ESM.docx]

**Additional File 1**

**Questionnaires**

Questions used in the study: the role of intention and self-efficacy on the association between breastfeeding of first and second child, a Danish cohort study

Six weeks postpartum, first child

Did your child receive supplement while you were in hospital?
 Yes No ❑ ❑

Did you receive the necessary support for breastfeeding?
 Yes Neither nor No
In the hospital ❑ ❑ ❑
From the health visitor ❑ ❑ ❑
From the child´s father ❑ ❑ ❑

One year postpartum, first child

How many weeks did you breastfeed your first child without giving any supplement at all?

(except from single occasions)
 -------------- weeks

How many weeks were your first child when you stopped breastfeeding entirely? -------------- weeks

How many weeks do you intend to breastfeed your next infant?
 -------------- weeks

How certain are you that you can carry on with breastfeeding your next infant until he/she reaches the age of four months?

Very certain ❑ Certain ❑

Neither nor ❑

Uncertain ❑

Very uncertain ❑

Five years postpartum, first child

Did your child receive supplement while you were in hospital?
 Yes No ❑ ❑

Did you receive the necessary support for breastfeeding:
 Yes Neither nor No
In the hospital ❑ ❑ ❑
From the health visitor ❑ ❑ ❑
From the child´s father ❑ ❑ ❑

How many weeks did you breastfeed your second child without giving any supplement at all? (except from on single occasions)
 -------------- weeks

How many weeks were your second child when you stopped breastfeeding entirely? -------------- weeks
